# *In Silico *profiling of deleterious amino acid substitutions of potential pathological importance in haemophlia A and haemophlia B

**DOI:** 10.1186/1423-0127-19-30

**Published:** 2012-03-16

**Authors:** George Priya Doss C

**Affiliations:** 1 Centre for Nanobiotechnology, Medical Biotechnology Division, School of Bio Sciences and Technology, VIT University, Vellore 632014, Tamil Nadu, India

**Keywords:** *In silico*, *F8*, *F9*, Haemophilia A, Haemophilia B

## Abstract

**Background:**

In this study, instead of current biochemical methods, the effects of deleterious amino acid substitutions in *F8 and F9 *gene upon protein structure and function were assayed by means of computational methods and information from the databases. Deleterious substitutions of *F8 and F9 *are responsible for Haemophilia A and Haemophilia B which is the most common genetic disease of coagulation disorders in blood. Yet, distinguishing deleterious variants of *F8 and F9 *from the massive amount of nonfunctional variants that occur within a single genome is a significant challenge.

**Methods:**

We performed an *in silico *analysis of deleterious mutations and their protein structure changes in order to analyze the correlation between mutation and disease. Deleterious nsSNPs were categorized based on empirical based and support vector machine based methods to predict the impact on protein functions. Furthermore, we modeled mutant proteins and compared them with the native protein for analysis of protein structure stability.

**Results:**

Out of 510 nsSNPs in *F8*, 378 nsSNPs (74%) were predicted to be 'intolerant' by SIFT, 371 nsSNPs (73%) were predicted to be 'damaging' by PolyPhen and 445 nsSNPs (87%) as 'less stable' by I-Mutant2.0. In *F9*, 129 nsSNPs (78%) were predicted to be intolerant by SIFT, 131 nsSNPs (79%) were predicted to be damaging by PolyPhen and 150 nsSNPs (90%) as less stable by I-Mutant2.0. Overall, we found that I-Mutant which emphasizes support vector machine based method outperformed SIFT and PolyPhen in prediction of deleterious nsSNPs in both *F8 *and *F9*.

**Conclusions:**

The models built in this work would be appropriate for predicting the deleterious amino acid substitutions and their functions in gene regulation which would be useful for further genotype-phenotype researches as well as the pharmacogenetics studies. These *in silico *tools, despite being helpful in providing information about the nature of mutations, may also function as a first-pass filter to determine the substitutions worth pursuing for further experimental research in other coagulation disorder causing genes.

## Background

Hereditary haemophilias are the most frequently encountered recessive inherited disease of coagulation disorders in blood. Haemophilia A and Haemophilia B are X-linked inherited bleeding disorder caused by a decreased activity or lack of coagulation factor VIII cofactor activity (haemophilia A) or coagulation factor IX enzyme activity (haemophilia B) due to heterogenous mutations in the *F8 and F9 *coding gene [1,2]. Factor VIII is a protein cofactor with no enzyme activity that, when activated, forms a complex with factor IXa serine protease on membrane surfaces. Upon activation, and in the presence of calcium ions and phospholipid surfaces, factor VIII and factor IX form an active complex, the tenase complex, which activates factor X during blood coagulation [3]. The *F8 *gene maps to the distal end of the long arm of X-chromosome (Xq28) and spans 186 kilo bases (kb) of genomic DNA. It consists of 26 exons and encodes a mature protein of 2,332 amino acids arranged within six domains organized as A1-A2-BA3-C1-C2 [4]. Its prevalence rate is estimated at 1:5,000-10,000 in men. Factor VIII circulates in the blood as a hetero dimer composed of two polypeptide chains: a light chain with a molecular weight of 80,000 Daltons (Da) and a heterogeneous heavy chain with a molecular weight varying between 90,000 and 200,000, Daltons (Da) both derived from the single peptide chain [5]. The *F9 *gene is much smaller than *F8 *maps to the distal end of the long arm of X-chromosome (Xq27) and spans 34 kb in length [6]. It contains 8 exons and encodes a glycoprotein of 415 amino acid residues, normally presents in plasma, which is an essential component of the clotting cascade [7]. It contains six major domains: signal peptide, propeptide, gla domain, two epidermal growth factor-like (EGF-like) domains, activation and catalytic domains [8]. The heterogeneous genetic diseases Haemophilia A & B, has been associated with missense mutations, nonsense mutations, gene deletions of varying size, insertions, inversions, and splice junction mutations and reported in Haemophilia A human database [9] and Haemophilia B human Database [2]. Classification of Haemophilia is based on plasma procoagulant levels, with persons with less than 1% active factor (< 0.01 IU/ml) are classified as having severe haemophilia, those with 1-5% active factor (0.01-0.05 IU/ml) have moderate haemophilia, and those with 5-40% of normal levels of active clotting factor (> 0.05- < 0.4 IU/ml) have mild haemophilia [10].

Recent advances in high-throughput genotyping and next generation sequencing have generated a tremendous amount of human genetic variation data, determining the effects of amino acid substitution will be the next challenge in mutation research. In the human genome single base substitutions called 'Single Nucleotide Polymorphisms' (SNPs) is the most frequent type of genetic variation. When SNPs occur in coding regions and produce amino acids change in the corresponding proteins, we name it as nonsynonymous single nucleotide polymorphisms (nsSNPs) [11]. Half of all genetic changes related to human diseases are attributed to nsSNP variants [12]. Differentiating deleterious nsSNPs (significant phenotypic consequences) from tolerant nsSNPs (without phenotypic change) are of great importance in understanding the genetic basis of haemophilias. This can be achieved by two general strategies: (i) carrier detection by linkage analysis (ii) *in silico *approach. Discriminating the types of nsSNPs in Mendelian disease genes, coupled with issues of statistical power, provide a compelling rationale for the application of a sequence-based approach to association studies rather than complete reliance on a map of anonymous haplotypes [13]. Because genome-wide scans are still financially challenging, it is advantageous to prioritize variants that may affect the structure or function of expressed proteins. NsSNPs can be analyzed according to the biochemical severity of the amino acid substitution and its context within the protein sequence. In this context, Grantham matrix [14] predicts the effect of amino acid substitutions based on chemical properties, including polarity and molecular volume. Recently more sophisticated *in silico *programs were developed and made available on the World Wide Web [15-23]. They take into account, to various degrees, factors such as the general rules of protein chemistry (e.g., change in charge or in hydrophobicity, or helix-breaking residue), the three dimensional structure of the protein, and homologies in amino acid sequences among various species or related proteins. These tools made use of a variety of features such as information derived from protein sequences or from both sequence and structural information. Deleterious nsSNPs analyses for the *F8 and F9 *genes have not been estimated computationally till now, although they have received great attention from experimental researchers. To answer this question, in the absence of other experimental investigations, we tested empirical rule based methods PolyPhen (Polymorphism Phenotyping) and Sorts Intolerant From Tolerant (SIFT) [11,20], machine-learning approach I-Mutant 2.0 [21], UTRScan [22] and PupaSuite [23] were used for prioritization of high-risk SNPs in coding and non-coding regions (5' and 3' un-translated regions (UTR) SNPs). Based on the scores of SIFT, PolyPhen and I-Mutant, we identified the deleterious nsSNPs that are likely to affect the protein structure. In order to understand the molecular mechanism of disease, it is important to determine the impact of these mutations on the structure. We have identified the potential mutations, proposed modeled structure for the mutant proteins, and compared them with the native protein. We also analyzed native and mutant modeled protein for stability analysis, solvent accessibility and secondary structure analysis.

## Methods

### Extraction of SNP information

The SNPs information (Protein accession number (NP), amino acid position, SNP ID, UniProtKB/Swiss-Prot source ID, and mRNA accession number (NM) of *F8 *and *F9 *was retrieved from the NCBI dbSNP [24] and SWISS-Prot databases [25] for our computational analysis. The information on the effect and the relationship between the nsSNPs and Haemophilia A and Haemophilia B disease was compiled from *in vivo *and *in vitro *experiments according to PubMed, OMIM [26], HAMSTeRS Database [9] and Haemophilia B [2] database and UniProtKB/Swiss-Prot databases.

### Assessment of nsSNP functionality

Empirical rules are derived based on sequence information, structural information or both. These methods predict deleterious nsSNPs based on physicochemical properties [14], protein structure [27-29], and cross species conservation [28-30]. The SIFT [20] and PolyPhen server [11] are the two representatives for this purpose.

### SIFT

SIFT program http://blocks.fhcrc.org/sift/SIFT.html uses "sequence homology to predict whether an amino acid substitution will affect protein function and hence, potentially alter phenotype". SIFT scores are classified as intolerant (0.00-0.05), potentially intolerant (0.051-0.10), borderline (0.101-0.20), or tolerant (0.201-1.00) [31,32]. The higher a tolerance index, the less functional impact a particular amino acid substitution is likely to have, as a higher tolerance index indicates that the position is less conserved across species.

### PolyPhen

PolyPhen a multiple sequence alignment server that aligns sequences using structural information. PolyPhen performs the prediction through sequence-based characterization of the substitution site, calculation of position-specific independent count (PSIC) profile scores for two amino acid variants, and calculation of structural parameters and contacts. The higher a PSIC score difference, the higher functional impact a particular amino acid substitution is likely to have. Predictions of how a particular nsSNP may affect protein structure by PolyPhen 2.0 are assigned as "probably damaging" a score (≥ 2.000) made with high confidence that the nsSNP should affect protein structure and/or function; "possibly damaging," score (1.500-1.999) where it may affect protein function and/or structure; and "benign," score (0.000-0.999) as most likely having no phenotypic effect.

### I-Mutant

I-Mutant 2.0 available at http://gpcr.biocomp.unibo.it/cgi/predictors/I-Mutant2.0/I-Mutant2.0.cgi. is a support vector machine (SVM)-based tool for the automatic prediction of protein stability changes and stabilization centers upon single point mutations. I-Mutant 2.0 predictions are performed starting either from the protein structure or, more importantly, from the protein sequence [21]. The output file shows the predicted free energy change value or sign (DDG) which is calculated from the unfolding Gibbs free energy value of the mutated protein minus the unfolding Gibbs free energy value of the native type (kcal/mol). If the DDG value is positive then the mutated protein will have high stability and vice versa for less stability. NsSNPs of *F8 *and *F9 *genes with experimental evidence of altered activity or disease association were considered as deleterious. The functional impact of the nsSNPs in *F8 *and *F9 *genes can be validated from the phenotypic data obtained from both *in vivo *and *in vitro *studies. Prediction accuracy of these computational methods was analyzed based on the positive findings from these benchmarking experiments obtained from HAMSTeRS Database and Haemophilia B database and UniProtKB/Swiss-Prot. nsSNPs predicted as "deleterious" and experimentally associated was considered as correct, while the prediction was defined as an error if such a deleterious nsSNP was predicted as tolerant. Concordance analysis between the functional consequences of each nsSNP of *F8 *and *F9 *genes predicted by SIFT, PolyPhen and I-Mutant were assessed using Spearman's rank correlation coefficient ρ.

### Predicting the molecular phenotypic effects of deleterious SNPs

The PupaSuite 3.1 [23] are now synchronized to provide annotations for both noncoding and coding SNPs, as well as annotations for the SwissProt set of human disease mutations. PupaSuite finds all the SNPs mapping in locations that might cause a loss of functionality in the genes. PupasView [33] retrieves SNPs that could affect conserved regions that the cellular machinery uses for the correct processing of genes (intron/exon boundaries or exonic splicing enhancers).

### Characterization of SNPs in regulatory untranslated regions

5' and 3' untranslated regions (UTR) of eukaryotic mRNAs are involved in many posttranscriptional regulatory pathways that control mRNA localization, stability and translation efficiency [34,35]. We used the program UTRScan http://itbtools.ba.itb.cnr.it/utrscan for this analysis. UTRScan looks for UTR functional elements by searching through user-submitted query sequences for the patterns defined in the UTRsite collection. Briefly, two or three sequences of each UTR SNP that have a different nucleotide at an SNP position are analyzed by UTRScan, which looks for UTR functional elements by searching through user-submitted sequence data for the patterns defined in the UTRsite and UTR databases. If different sequences for each UTR SNP are found to have different functional patterns, this UTR SNP is predicted to have functional significance.

### Modeling nsSNP Locations on FVII and FIX

Structural analysis was performed based on the crystal structure of the protein for evaluating the structural stability of native and mutant protein. We used the SAAPdb [36] and dbSNP to identify the protein coded by *F8 *gene with PDB ID 2R7E[37] and *F9 *gene with PDB ID 1RFN[38]. We also confirmed the mutation positions and the mutation residues from this server. These mutation positions and residues were in complete agreement with the results obtained with SIFT and PolyPhen programs. Based on the position of amino acids in the corresponding chains of the crystallized structures, the mutation analysis was performed using SWISSPDB viewer [39], and energy minimization was carried out using the program package GROMACS 4.0.5 [40] with Force field GROMOS96 43a1 [41]. The native and mutant proteins were solvated in cubic 0.9 nm of simple point charge (SPC) water molecules [42]. A periodic boundary condition was applied that the number of particles, pressure, and temperature was kept constant in the system. The system was neutralized by adding Na^+ ^and Cl^- ^ions around the molecules to obtain electrically neutral system. The native and mutant structures were first minimized with steepest descent by 2000 steps and conjugated gradient by 3000. Computing total energy gives information about the protein structure stability. The deviation between the two structures is evaluated by their RMSD (root mean square deviation) values which could affect stability and functional activity [43]. By visualizing the position of the mutated amino acid residues; it is possible to suggest a physiochemical rationale for the effect on protein activity. The quality of 3D structure was assessed two programs: Verify 3D [44,45] and Prosa-Web [46].

### Analyzing the effects of mutations on protein stability

We obtained the solvent accessibility information using program GETAREA [47] available at http://curie.utmb.edu/getarea.html. For a successful analysis of the relation between amino acid sequence and protein structure, an unambiguous and physically meaningful definition of secondary structure is essential. We obtained the information about secondary structures of the proteins using the program DSSP [48]. The prediction of solvent accessibility and secondary structure has been studied as an intermediate level for predicting the tertiary structure of proteins.

## Results

### Predictions of deleterious and damaging coding nsSNPs

SNPs information for *F8 *and *F9 *was retrieved from dbSNP and cross verified with Swiss-prot database. For our investigations, we selected SNPs in nsSNPs and UTR (5'and 3') regions. Among the 675 nsSNPs, 510 nsSNPs (177 RefSNPs and 333 Swiss-Prot SNPs) and 165 nsSNPs (67 RefSNPs and 98 Swiss-Prot SNPs) were in *F8 *and *F9*, 17 and 9 SNPs in mRNA of *F8 *and *F9 *were in included in our analysis. We applied three *in silico *tools SIFT, PolyPhen and I-Mutant 2.0 to predict the putative effect of each nsSNP on protein function.

### F8

The protein sequences of 510 nsSNPs were submitted separately to the SIFT program to inspect its tolerance index. We identified a total of 378 nsSNPs (74%) that were scored as intolerant by SIFT. Approximately 197 nsSNPs (39%) exhibited SIFT scores of 0.0; 181 nsSNPs (35%) showed scores between 0.01-0.05; 9% of the variants (45 nsSNPs) have scores between 0.051-0.10 and the remaining 17% of the nsSNPs were classified as 'Tolerant' by SIFT. PolyPhen identified a total of 371 nsSNPs (73%) that were scored as damaging. 183 nsSNPs (36%) exhibited PolyPhen score of > 2.00, 188 nsSNPs (37%) have scores between 1.99-1.50, and 70 nsSNPs (13.5%) have scores between 1.49-1.25. Consequently, 69 nsSNPs (13.5%) were characterized as benign. We analyzed the nsSNPs finally by using I-Mutant server. 445 nsSNPs (87%) were found to be less stable and exhibited a DDG value ranging from -0.02 to -5.23 respectively. Approximately 296 nsSNPs (58%) showed a DDG value of ≤ -1.0; 169 nsSNPs (33%) showed a DDG value -1.01 to -2.00 and the remaining 45 nsSNPs (9%) showed a DDG value ≥ -2.01 respectively. Additional file 1: Table S1 represents the distribution of nsSNPs by SIFT, PolyPhen, and I-Mutant scores.

### F9

Similarly, a total of 165 nsSNPs in *F9 *gene were submitted to SIFT, PolyPhen and I-Mutant 2.0. Additional file 1: Table S1 represents the distribution of nsSNPs by SIFT PolyPhen and I-Mutant 2.0 scores. By SIFT, 90 nsSNPs (55%) showed a tolerance index score of 0.00; 39 nsSNPs (24%) showed scores between 0.01-0.05; 7 nsSNPs (4%) showed scores between 0.051-0.10 and the remaining 29 nsSNPs (17%) were classified as 'Tolerant' by SIFT. When PolyPhen was applied for prediction, 105 nsSNPs (64%) were scored as > 2.00; 26 nsSNPs (16%) exhibited scores between 1.99-1.50, 8 nsSNPs (4.5%) have scores between 1.49-1.25. Consequently, 69 nsSNPs (15.5%) were characterized as benign. By I-Mutant, 82 nsSNPs (49.6%) showed a DDG value of ≤ -1.0; 55 nsSNPs (33%) showed a DDG value -1.01 to -2.00, 13 nsSNPs (7.9%) showed a DDG value ≥ -2.01, 15 nsSNPs (9.1%) showed a DDG value < 0.00 respectively.

### Correlation of computational methods in prediction of nsSNPs in haemophliacs

We combined scores of different prediction programs SIFT, PolyPhen, and I-Mutant 2.0 and found that this could significantly increase prediction performance nsSNPs analysis (Table 1). There are evidences to state combinatorial approach using different computational methods performed well in increasing the accuracy in prediction of functional and deleterious nsSNPs [49]. Since a lower SIFT or I-Mutant 2.0 score indicate that the nsSNPs of interest would be more deleterious, whereas a higher PolyPhen score indicate that the nsSNPs of interest would be more deleterious. Among 510 nsSNPs in *F8*, 378 nsSNPs (74%) were predicted to be intolerant by SIFT, 371 nsSNPs (73%) were predicted to be damaging by PolyPhen and 445 nsSNPs (87%) as less stable by I-Mutant2.0. In *F9*, 129 nsSNPs (78%) were predicted to be intolerant by SIFT, 131 nsSNPs (79%) were predicted to be damaging by PolyPhen and 150 nsSNPs (90%) as less stable by I-Mutant2.0. By our analysis we found that I-Mutant outperformed SIFT and PolyPhen in prediction of deleterious nsSNPs in both *F8 *and *F9*. Most of these differences are likely the result of each method requiring a sufficient number and diversity of aligned sequences in order to make a prediction, each method using a different set of sequences and alignments. Our earlier analysis also shown individual tools correlate modestly with observed results, and that combining information from different tools may perform better in increasing the predictive accuracy in determining the functional impact of a given nsSNP [49]. In combination the nsSNPs which were predicted to be deleterious in causing an effect in the structure and function of the protein by SIFT, PolyPhen, and I-Mutant 2.0 correlated well experimental studies as shown in Table S1 [50-130].

**Table 1:** Concordance analysis between SIFT and PolyPhen in the prediction of functional variants in *F8 *and *F9*

SIFT		No of Variants		PolyPhen		No of Variants	
**Scores**	**Prediction**	***F8***	***F9***	**Scores**	**Prediction**	***F8***	***F9***

0.00-0.05	Intolerant	378	129	≥ 2.000	Probably damaging	183	105

0.051-0.10	Potentially intolerant	45	7	1.500-1.999	Possibly damaging	188	26

0.101-0.20	Borderline	43	8	1.25-1.49	Potentially damaging	70	8

0.201-1.00	Tolerant	44	21	1.00-1.24	Borderline	49	3

				0.000-0.999	Benign	20	23
				
**Total**		**510**	**165**	**Total**		**510**	**165**

### Predictions of potential phenotypic effect in SNPs

Among 29 SNPs predicted by PupaSuite, 26 nsSNPs were found to disrupt Exon Splicing Enhancer and 3 nsSNPs were predicted to disrupt Exon Splicing Silencer as depicted in Additional file 1: Table S1 respectively. Four SNPs namely rs1803603, rs34683807, rs1396947, and rs5986887 were predicted to disrupt Exon Splicing Enhancer and SNP namely rs34700571 was predicted to disrupt Exon Splicing Silencer in untranslated region of *F8 *gene.

### Functional SNPs in non-coding SNPs

Polymorphism in the 3'UTR region affects the gene expression by affecting the ribosomal translation of mRNA or by influencing the RNA half-life. UTResource was applied to prioritize 17 SNPs (*F8*) and 9 SNPs in (*F9*) UTR region. After comparing the functional elements for each UTR SNP, we found that only 5 SNPs were predicted to have functional significance. Four SNPs namely rs36101366, rs34683807, rs5986887, rs1396947 were related to functional pattern change of Upstream Open Reading Frame (UOF) and rs4487960 related to Polyadenylation Signal Upstream Open Reading Frame (uORF) in *F8*, and two SNPs with ID rs191483077 and rs186616567 were related to functional pattern change of Internal Ribosome Entry Site (IRES) in *F9 *respectively.

### Structural analysis

**Figure 1:**
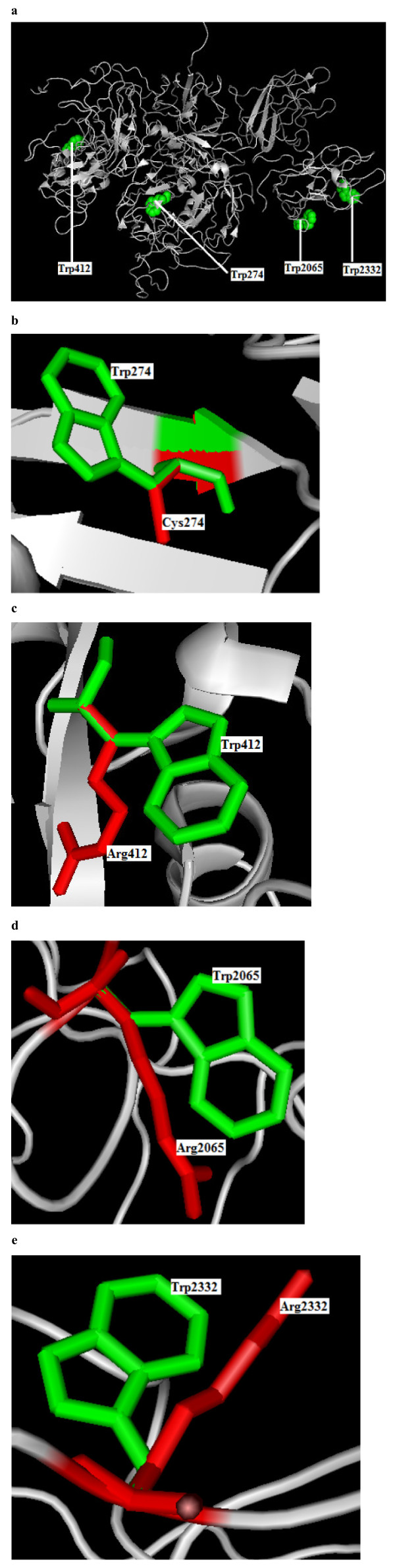
**Structural representation of FVIII (2R7E) native and mutant proteins**. **a**. Structure of FVIII native type protein (2R7E) in grey displaying the position of W274, W412, W2065 and W2232 in sphere shape (green color). **b**. Superimposed structure of native amino acid tryptophan in sphere shape (green color) with mutant amino acid cysteine (red color) at position 274 in ‘A’ chain of 2R7E. **c**. Superimposed structure of native amino acid tryptophan in sphere shape (green color) with mutant amino acid arginine (red color) at position 412 in ‘A’ chain of 2R7E. **d**. Superimposed structure of native amino acid tryptophan in sphere shape (green color) with mutant amino acid arginine (red color) at position 2065 in ‘B’ chain of 2R7E. **e**. Superimposed structure of native amino acid tryptophan in sphere shape (green color) with mutant amino acid arginine (red color) at position 22232 in ‘B’ chain of 2R7E.

**Figure 2:**
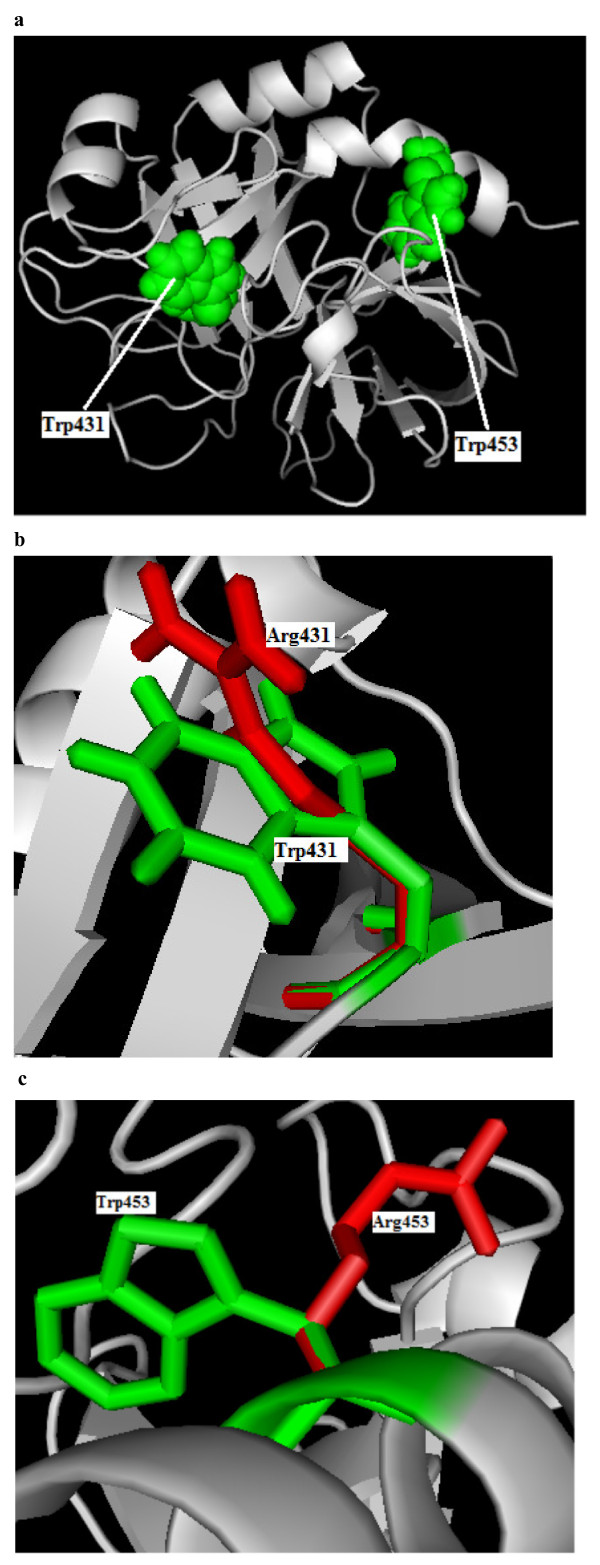
**Structural representation of FIX (2WPH) native and mutant proteins**. **a**. Structure of FIX native type protein (2WPH) in grey displaying the position of W431 and W453 in sphere shape (green color). **b**. Superimposed structure of native amino acid tryptophan in sphere shape (green color) with mutant amino acid arginine (red color) at position 412 in ‘S’ chain of 2WPH. **c**. Superimposed structure of native amino acid tryptophan in sphere shape (green color) with mutant amino acid arginine (red color) at position 453 in ‘S’ chain of 2WPH.

Single amino acid mutations can significantly alter the stability of a protein structure. So, the knowledge of a protein's three-dimensional (3D) structure is essential for a full understanding of its functionality. Mapping the deleterious nsSNPs into protein structure information was obtained from dbSNP and SAAPdb. Available X-ray crystallized structures for the FVIII and FIX protein in Protein Data Bank with PDB ID code 2R7E (3.70 Å), and 2WPH (1.5 Å). Mutation analysis was performed based on the results obtained from highest SIFT, and PolyPhen scores. It is noted that rs34371500 (W274C), VAR_028524 (W412R) in 'A' chain and rs28937299/rs137852455 (W2065R), and VAR_028712 (W2332R) in 'B' chain of PDB ID 2R7E, showed the highest deleterious score of 0.00 (SIFT) and damaging scores (PolyPhen) ranging from 3.318 to 3.543 respectively in FVIII. Similarly in FIX, VAR_006611 (W431R), and rs137852269 (W453R) showed the highest deleterious score of 0.00 (SIFT) and damaging scores (PolyPhen) of 4.434 and 4.632 respectively. For W431R and W453R mutation analysis was performed in the 'S' chain of the PDB ID 2WPH. The mutations for FVIII and FIX at their corresponding positions were performed by SWISS-PDB viewer independently to achieve modeled structures. Then, energy minimizations were performed by GROMACS 4.0.5 for the native type protein and the mutant type structures. Total energy and the RMSD values between the native (2R7E and 2WPH) and the mutant amino acids were calculated. Higher the RMSD value more will be the deviation between native and mutant type structures and which in turn changes their functional activity. In this analysis found that the total energy for the mutant proteins W274C, W412R, W2065R, and W2332R following energy minimization was -97899.13, -98142.42, -98013.21 and -97013.21 kJ/mol when compared to native protein (2R7E) energy -98911.33 kJ/mol. The RMSD values were calculated between the native and mutant amino acids and showed 2.74 Å in W274C, 2.78 Å in W412R, 2.85 Å in W2065R and 2.91 Å in W2332R. The superimposed structures of the native protein with the four mutant type proteins are shown in Figure 1a-d respectively. These figures were drawn using PyMOL54 release 0.99 [131]. Similarly, we checked the total energy for mutant type structure W431R and W453R were found to be -81428.83 and -81694.21 when compared to native energy of -84591.35 kJ/mol. The RMSD values were calculated between the native and mutant amino acids and showed 2.94 Å in W431R, and 3.18 Å in W453R. The superimposed structures of the native protein with the four mutant type proteins are shown in Figure 2a-c, respectively. In W412R, W2065R, W2332R W431R and W453R there is change in drift of charge from non-polar to polar residue Substitution of positively charged arginine in place of neutral tryphtophan may lead to disturbance in the interactions with other molecules or other parts of the proteins. These types of substitutions could introduce repulsive interactions between neighboring residues. Similarly we observed the potential effects of substitutions such as disruption of ligand binding site, disruption of annotated functional site, overpacking at buried site, contact with functional site, and hydrophobicity change at buried site in PolyPhen predictions. Solvent accessibilities and secondary structures of amino acid residues in the native and mutant proteins were analyzed by GETAREA and DSSP as shown in Table 2.

**Table 2:** Solvent accessibilities and Secondary structure analysis in the native and mutant proteins

Mutation Position	Solvent accessibility in the native and mutant proteins by GETAREA		Secondary structure analysis by DSSP
		
	Changed from exposed to buried	Changed from buried to exposed	
W274C	Thr (2), Tyr(5) Leu(7), Val(63), His(76), Ala(81), Pro(93), Ser(116), Ala(119), Glu(129), Lys(146), Tyr(155), Lys(185), Leu(187), Ala(194), Lys(213), Leu (217), Ile(310), Leu(319), Leu(327), Gln(335), Glu(340), Lys(344), Pro(349), Lys(399), Thr(400), Leu(419), Tyr(426), Lys(431), Lys(441), Ala(449), Thr(454), Lys(485), Arg(509), Gly(539), Thr(549), Ser(553), Glu(559), Glu(576), Asp(579), Gln(585), Lys(589), Val(592), Phe(598), Arg(602), Leu(614), Asn(631), Tyr(639), Ser(647), Ala(654), His(679), Leu(689), Val(697), Asn(713), Arg(719), Ser(728), Lys(732)	Trp(14), Leu(69), Gln(96), Val(99), Ser(138), Ser(176), Ser(179), Ile(405), Asn(299), Cys(329), Val(392), Arg(424), Tyr(473), Arg(503), Arg(546), Val(556), Pro(569), Gly(565), Asn(609), Gly(619), Ile(636), Trp(707)	T → H: Pro(86), Gly(89), Leu(203), Thr(208), Ser(428), Lys(518), Gly(638), His(1735), Thr(1763), Lys(1932), Asn(1934), Met(1945), Glu(2200) H → T:Lys(161), Lys(185), Leu(187), Leu(562), Asn(601), Glu(608), Arg(612), Ser(630), Asp(1865), Gly(2022) T → S:Trp(87), Leu(517), Asn(637), Asn(1772)

W2065R	Asp(1260), Gln(1336), Leu(1481), Ala(1610), Ile(1698), Tyr(1699), Arg(1708), Thr(1714), Glu(1723), Arg(1740), Gly(1769), Leu(1775), Ile(1782), Arg(1800), His(1867), Leu(1882), Trp(1908), Ala(1939), Asn(1941), Met(1945), Arg(1960), Ser(1968), Asn(1971), Phe(1982), Met(2007), Arg(2016), Ser(2082), Arg(2169)	Asn(1460), Gln(1705), Gly(1779), Leu(1808), Val(1876), Glu(1884), Glu(1904), Met(1842), Ile(1901), Tyr(1909), Thr(2015),	T → H:), His(1735), Thr(1763), Lys(1932), Asn(1934), Met(1945), Glu(2200) H → T: Asp(1865), His(1867), Asp(2206) T → S: Asn(1772)

W2248C	Asp(1260), Gln(1336), Asn(1460), Leu(1481), Ala(1610), Ile(1698), Tyr(1699), Arg(1708), Thr(1714), Glu(1723), Arg(1740), Gly(1769), Leu(1775), Ile(1782), Arg(1800), His(1867), Val(1876), Leu(1882), Trp(1908), Ala(1939), Asn(1941), Met(1945), Arg(1960), Ser(1968), Asn(1971), Phe(1982), Met(2007), Arg(2016), Gly(2028), Ala (2070)Ser(2082), Arg(2169)	Asn(1460), Gln(1705), Gly(1779), Leu(1808), Val(1876), Glu(1884), Glu(1904), Met(1842), Ile(1901), Tyr(1909), Thr(2015), Gly(2022), Ala(2070)	T → H:), His(1735), Thr(1763), Lys(1932), Asn(1934), Met(1945), Glu(2200) H → T: Asp(1865), Gly(2022)

T-Turn, H-Helix, S-Strand

## Discussion

Predicting phenotypic consequences of nsSNPs by application of bioinformatics analysis may provide a good way to explore the function of nsSNPs and the relationship between nsSNPs and susceptibility to disease. The mutations causing haemophilia A and B have been localized and well characterized by several experimental studies. Most of the mutations in haemophilias leads to insufficient activity of the tenase complex, brought about either by a deficiency of coagulation factor VIII cofactor activity (haemophilia A) or coagulation factor IX enzyme activity (haemophilia B). Thus, it is not surprising that the two disorders are clinically similar because they both arise from perturbation of the same essential step in the process of fibrin generation. It is also clearly evident the molecular basis of the haemophilias is extremely diverse from the enormous number of mutations that have been elucidated so far. For this purpose, vast number of bioinformatic tools, based on recent findings from evolutionary biology (amino acid sequence), protein structure analysis, and computational biology may provide useful information in assessing the functional significance of SNPs have been proposed. In this study, we explored the relationship between prediction consequences of nsSNPs by computational approaches based on recent findings from evolutionary biology, protein structure research, and real phenotypes confirmed by experiments. The recent progress made in experimental 3D structure determination of FVIII and FIX by X- ray crystallography [37,38] and modeling studies [132] have made it possible to predict the effects of nsSNPs at structural level by mapping them on corresponding structures. The functional consequences of most SNPs *F8 *and *F9 *gene are still unknown, although some nsSNPs have been associated with X-linked inherited bleeding disorder. *In vivo and in vitro *studies on the function of nsSNPs have found that genetic mutations in *F8 *and *F9 *gene are responsible for Haemohpilia A and Haemohpilia B. There have been a quite lot of studies existing to validate the importance of single amino acid substitutions in Haemophlia A and Haemophilia B at activation cleavage sites [65,133,134], affecting factor VIII binding to von Willebrand factor [65,101,135] factor VIII secretion [92] and factor IX binding to factor VIII [136-138]. Recently Markoff by his homology modeling approach analyzed the impact of substitutions in loss of S-S bridges, thrombin activation site, gain/loss of H-bonds, cross-chain H-bonds, ionic bond and possible contact residue in FVIII [132]. The information regarding the involvement of mutations in Gla (g-carboxyglutamic acid) domain, EGF1 for the N-terminal domain that binds calcium, EGF2 where it does not bind calcium, and a catalytic serine protease (SP) domain has been deposited in CoagMDB database [138]. The most commonly observed amino acid substitutions are Arg, Tyr, Phe and Cys in FVIII and FIX which play important role in altering the protein function. These amino acids are involved in protein folding dependent on disulfide bonds (Cys) and protein active or binding sites (Arg) [139,140]. These amino acids predicted deleterious by SIFT, PolyPhen and I-Mutant were in concordance with the experimental studies (Additional file 1: Table S1). It is becoming clear that implementation of the molecular evolutionary approach may be a powerful tool for prioritizing SNPs to be genotyped in future molecular epidemiological studies. Moreover, from an evolutionary perspective, SNPs altering a conserved amino acid site are more likely to have functional importance. Computational tools like SIFT and PolyPhen are able to predict 90% of damaging SNPs. Several groups also validated these algorithms has, however, come from benchmarking studies based on the analysis of "known" deleterious substitutions annotated in databases, such as SwissProt. In such studies, PolyPhen and SIFT has been shown to successfully predict the effect of over 80% of amino acid substitutions [31,32,141]. In this study, we first surveyed previous publications and submitted mutations in database associated with Haemophilia A and Haemophilia B, the most extensively examined bleeding disorder. In this present study three different widely-used computational tools were employed for determining the functional significance of nsSNPs. First, we included functional scores from SIFT, PolyPhen and I-Mutant tools, each of which employs fundamentally different algorithms that can be used to determine the functionality of the same nsSNPs. Proteins with mutations do not always have 3D structures that are solved and deposited in PDB. Therefore, it is necessary to construct 3D models using homology modeling by locating the variation in 3D. This is a simple way of detecting what kind of adverse effects that a mutation can have on a protein.

Based on the SIFT, PolyPhen, and I-Mutant scores and availability of 3D structures, structure analysis was carried out with the major mutation that occurred in the native protein coded by *F8 *and *F9*. The total energy and RMSD value of mutant structures W274C, W412R, W2065R, W2332R, W431R, and W453R were calculated. Correlations between SIFT (Sensitivity 83%), PolyPhen (Sensitivity 82%) and I Mutant (Sensitivity 80%) were calculated from raw scores rather than the arbitrarily defined categories. There was a significant correlation between the predictions obtained using SIFT and PolyPhen algorithms in both *F8 *(*ρ *= -0.59) and *F9 *(*ρ *= -0.576); while the correlation between the predictions obtained using PolyPhen and I Mutant in *F8 *(*ρ *= -0.86) and *F9 *(*ρ *= -0.63) were much higher. A positive correlation was observed with SIFT and I- Mutant score for *F8 *(*ρ *= 0.30) and *F9 *(*ρ *= 0.31). We have shown that our data suggests that different tools correlate modestly with observed results, and that combining information from a variety of tools may significantly increase the predictive power for determining the functional impact of a given nsSNP.

## Conclusion

In conclusion, from our *in silico *analysis it is very difficult to determine whether the notable differences exists in the performance of these methods in predicting deleterious nsSNPs in *F8 *and *F9*. The variation in the prediction might be due to the difference in features utilized by the methods or the training datasets. There is no single literature stating a single *in silico *method can aid in better prediction. Our *in silico *analysis coincides with previous analysis performed by other groups stating that combining information obtained from various methods can increase prediction performance. The overall strategy of our study was to prioritize the functional nsSNPs, map as many structural mutations as possible, find general patterns to analyze 3D mutations with respect to protein function and evaluate regulatory variants using many *in silico *analysis methods as possible. Based on these analyses, we try to determine the relationship between the disease-related mutations and structural properties of proteins in haemophiliacs.

## Abbreviations

ESE: Exon splicing enhancer; ESS: Exon splicing silencer; NCBI: National center for biotechnology Information; nsSNPs: Non-synonymous single nucleotide Polymorphisms; OMIM: Online mendelian inheritance in man; PSIC: Position specific independent count; RMSD: Root mean square deviation; SIFT: Sorting intolerance from tolerance; SNP: Single nucleotide polymorphisms; UTR: Untranslated region.

## Competing interests

The author declares that they have no competing interests.

## Authors' contributions

CGPD collected the SNP data from the databases, analyzed the SNPs using different algorithms, predicted and the deleterious SNPs. CGPD also carried out the modeling analysis and drafted the manuscript.

## Supplementary Material

Additional file 1 **Table S1**. List of nsSNPs found to be functionally significant by SIFT, PolyPhen, I-Mutant and PupaSuite. Click here for file
