# Supplementary material for: In Silico profiling of deleterious amino acid substitutions of potential pathological importance in haemophlia A and haemophlia B
Source: J Biomed Sci. 2012 Mar 16;19(1):30. doi: 10.1186/1423-0127-19-30 (PMC3361463; doi:10.1186/1423-0127-19-30)
Supplement: Additional file 1 — Table S1. List of nsSNPs found to be functionally significant by SIFT, PolyPhen, I-Mutant and PupaSuite. [file 1423-0127-19-30-S1.DOC]

**Figure Legends**

**Fig. 1a**.Structure of FVIII native type protein (2R7E) in grey displaying the position of W274, W412, W2065 and W2232 in sphere shape (green color)

**Fig. 1b**. Superimposed structure of native amino acid tryptophan in sphere shape (green color) with mutant amino acid cysteine (red color) at position 274 in ‘A’ chain of 2R7E.

**Fig. 1c**. Superimposed structure of native amino acid tryptophan in sphere shape (green color) with mutant amino acid arginine (red color) at position 412 in ‘A’ chain of 2R7E.

**Fig. 1d**. Superimposed structure of native amino acid tryptophan in sphere shape (green color) with mutant amino acid arginine (red color) at position 2065 in ‘B’ chain of 2R7E.

**Fig. 1e**. Superimposed structure of native amino acid tryptophan in sphere shape (green color) with mutant amino acid arginine (red color) at position 22232 in ‘B’ chain of 2R7E.

**Fig. 2a**.Structure of FIX native type protein (2WPH) in grey displaying the position of W431 and W453 in sphere shape (green color).

**Fig. 2b**. Superimposed structure of native amino acid tryptophan in sphere shape (green color) with mutant amino acid arginine (red color) at position 412 in ‘S’ chain of 2WPH.

**Fig. 2c**. Superimposed structure of native amino acid tryptophan in sphere shape (green color) with mutant amino acid arginine (red color) at position 453 in ‘S’ chain of 2WPH.
